# Targeted Arginine Metabolism Therapy: A Dilemma in Glioma Treatment

**DOI:** 10.3389/fonc.2022.938847

**Published:** 2022-07-11

**Authors:** Xiaoshuang Hou, Sui Chen, Po Zhang, Dongsheng Guo, Baofeng Wang

**Affiliations:** Department of Neurosurgery, Tongji Hospital, Tongji Medical College, Huazhong University of Science and Technology, Wuhan, China

**Keywords:** glioma, arginine metabolism, T lymphocytes, tumor microenvironment, metabolic reprogramming

## Abstract

Efforts in the treatment of glioma which is the most common primary malignant tumor of the central nervous system, have not shown satisfactory results despite a comprehensive treatment model that combines various treatment methods, including immunotherapy. Cellular metabolism is a determinant of the viability and function of cancer cells as well as immune cells, and the interplay of immune regulation and metabolic reprogramming in tumors has become an active area of research in recent years. From the perspective of metabolism and immunity in the glioma microenvironment, we elaborated on arginine metabolic reprogramming in glioma cells, which leads to a decrease in arginine levels in the tumor microenvironment. Reduced arginine availability significantly inhibits the proliferation, activation, and function of T cells, thereby promoting the establishment of an immunosuppressive microenvironment. Therefore, replenishment of arginine levels to enhance the anti-tumor activity of T cells is a promising strategy for the treatment of glioma. However, due to the lack of expression of argininosuccinate synthase, gliomas are unable to synthesize arginine; thus, they are highly dependent on the availability of arginine in the extracellular environment. This metabolic weakness of glioma has been utilized by researchers to develop arginine deprivation therapy, which ‘starves’ tumor cells by consuming large amounts of arginine in circulation. Although it has shown good results, this treatment modality that targets arginine metabolism in glioma is controversial. Exploiting a suitable strategy that can not only enhance the antitumor immune response, but also “starve” tumor cells by regulating arginine metabolism to cure glioma will be promising.

## Introduction

Glioma is the most common primary malignant tumor of the central nervous system (CNS), accounting for 48% of all primary malignant CNS tumors (1); the most malignant type of glioma is glioblastoma (GBM). Although various treatment modalities including surgery, radiotherapy, chemotherapy, tumor treatment fields, molecular targeted therapy as well as supportive care have been employed in the treatment of GBM, the median survival time of the patients is less than two years, and the 5-year survival rate is less than 10% (2). The main reasons for the poor prognosis of patients with GBM are tumor infiltration, recurrence, and resistance to conventional therapy, which are closely related to intra-tumoral heterogeneity and phenotypic plasticity in GBM (3).

The CNS was considered an immune-privileged organ. However, this dogma was broken with the discovery that lymphatic vessels exist in the CNS (4) and that immune cells can cross the blood-brain barrier (BBB) (5). Many innate and acquired immune cells reside in the boundary zones of the CNS (6, 7). Different from the brain parenchyma, there is a large amount of lymphocyte infiltration that mediates the immune response (8). The lymphatic system in the meninges, and the cerebrospinal fluid, and the lymphocytes present in the meninges form a relatively mature network. This network allows antigens in the cerebrospinal fluid to enter the lymphatic system through the cervical lymph nodes, thereby initiating the activation of T cells (9, 10). This process has been confirmed in various diseases, including GBM. However, in pathological conditions such as GBM, the blood-brain barrier is destroyed; this results in increased permeability. Leukocytes, including antigen-presenting cells, enter the CNS through the choroid plexus, meningeal barrier, and postcapillary venules (5, 11), leading to infiltration of immune cells into tumor tissues (12, 13). GBM is a “cold” tumor owing to a lack of lymphocyte infiltration (14). The immune cells that infiltrate GBM are mainly macrophages and lymphocytes, such as CD4+ and CD8+ T cells; the concentration of T lymphocytes is positively correlated with the survival time of patients (15).

Advances in immunotherapy, such as the use of immune checkpoint inhibitors, have revolutionized cancer therapy. Unfortunately, these have been unsuccessful in the treatment of GBM (14, 16). The main obstacle in the treatment of GBM is the heterogenous and immunosuppressed tumor microenvironment, which results partly due to altered cellular metabolism (17). Cellular metabolism has become a determinant of the viability and function of cancer cells as well as immune cells. Tumors are metabolically reprogrammed to maintain enormous anabolic demands, which leads to the development of a microenvironment that is acidic, hypoxic, and devoid of the key nutrients required by immune cells. In this context, tumor metabolism is a checkpoint because it mediates tumor immune escape (18). The interplay between immune regulation and metabolic reprogramming in GBM is an active and stimulating area of research (18, 19). For example, enhanced glycolysis results in a glucose-starved microenvironment that makes tumors more aggressive. Glucose is a key nutrient that supports the rapid and dynamic transition of immune cells from the naïve state to an activated state (20). Reprogramming of amino acid metabolism in tumors often involves nutritional competition between cancer and immune cells. A large number of basic and clinical studies have shown that the use of new drugs that target tumor-dependent amino acid metabolism can effectively inhibit tumor growth. We noticed that arginine in the GBM microenvironment may be associated with the antitumor function of T lymphocytes.

Arginine promotes a series of metabolic reactions, including the synthesis of nitric oxide, polyamines, glutamine, and proline, all of which are important regulators of cell growth and survival (21). Arginine also exerts an essential regulatory effect on the immune system. Arginine-deficient T cells exhibit cell cycle arrest, impaired proliferation, reduced activation, and reduced antitumor activity (22–25). The reprogramming of arginine metabolism in GBM includes upregulation of the expression of amino acid transporters for intake of arginine, upregulation of the expression of arginase to decompose arginine, and downregulation of the expression of key enzymes involved in the endogenous arginine synthesis pathway. The former causes a deficit of arginine in the microenvironment, thereby inhibiting the function of T lymphocytes and promoting the formation of an immunosuppressive microenvironment. The latter represents a defect in cancer cell metabolism, and targeting this metabolic defect is a strategy used for treating tumors. Since the rate of proliferation of cancer cells is much higher, they require more nutrients, which exceeds their ability to synthesize amino acids (26). Cancer cells are dependent on extracellular arginine because of the decreased expression of arginine-synthesizing enzymes, argininosuccinate synthase (ASS1) and argininosuccinate lyase (ASL). In the absence of extracellular arginine, cancer cells become arginine dystrophic, or “arginine auxotrophic” (27). This strategy has been successfully used to treat acute lymphoblastic leukemia, in which asparaginase combined with chemotherapy has become the standard treatment (28). Mycoplasma infection was initially found to kill cancer cells (29). It was subsequently found that this is due to arginine deaminase (ADI), which degrades arginine in Mycoplasma (30, 31). Researchers then began using arginine deaminase and another enzyme, arginase (ARG), to break down arginine for the treatment of various tumors, including gliomas. Extensive preclinical and clinical research is being conducted on arginine deprivation therapy (32).

In this review, we describe how the unique metabolism of arginine in the glioma microenvironment leads to the suppression of the antitumor activity of T lymphocytes, thereby leading to tumor immune escape. We also discuss how targeting arginine metabolism in gliomas not only inhibits tumor growth, but also promotes effective and durable antitumor immunity.

## Metabolism of Arginine

### Arginine Metabolism in Humans

Arginine is a semi-essential amino acid that is found in adults. The humans can synthesize arginine, but under certain physiological stresses, such as burns or severe immune challenges, the humans needs to supplement dietary arginine (33–36). Arginine in adult circulation has a short half-life (37). Plasma arginine concentration is regulated by dietary arginine intake, endogenous arginine synthesis, arginine catabolism, hepatic urea cycle, and protein synthesis. It is important to note that changes in the dietary intake of arginine do not alter the rate of its endogenous synthesis, which lays the foundation for targeting arginine metabolism for the treatment of some specific diseases (38). Endogenous arginine is mainly synthesized through the intestinal–renal axis (39, 40). Although the urea cycle in the liver can synthesize arginine, there is very little net arginine synthesis in the liver (41, 42).

CNS tumors, such as gliomas, require more arginine; however, the CNS cannot increase the synthesis of arginine to meet the needs of cancer cells, and it can only increase arginine intake from the blood (43). For infiltrating immune cells, macrophages can express both ASS1 and ASL to synthesize arginine from citrulline (44, 45), which may be related to the fact that macrophages can account for 30%-50% of cells in the glioma microenvironment (46). However, not all immune cells simultaneously express all the enzymes required for *de novo* synthesis of arginine. For example, T cells rely only on a circulating supply of arginine or its immediate precursor.

In addition to protein synthesis, arginine has multiple functions such as vasodilation, neurotransmission, cell proliferation, and immune regulation (47, 48). The effect of arginine on the immune system has been gradually discovered in the last century. In 1968, the inhibitory effect of arginine deficiency on T lymphocyte activation *in vitro* was first described (49). Clinically, arginine is required for wound healing (50–52). Immune-enhancing diets (IED) use dietary arginine to stimulate the immune system (53, 54). These diets contain two to six times the arginine content of a normal diet. IEDs can boost immunity in trauma patients and reduce infection risk in surgical patients (55–57). It is important to note that IEDs do not benefit all patients (58). Determining whether arginine metabolism modulates immune cell function in specific diseases will undoubtedly lead to the development of more efficient individualized treatments.

### Metabolism of Arginine in Cells

**Figure 1:**
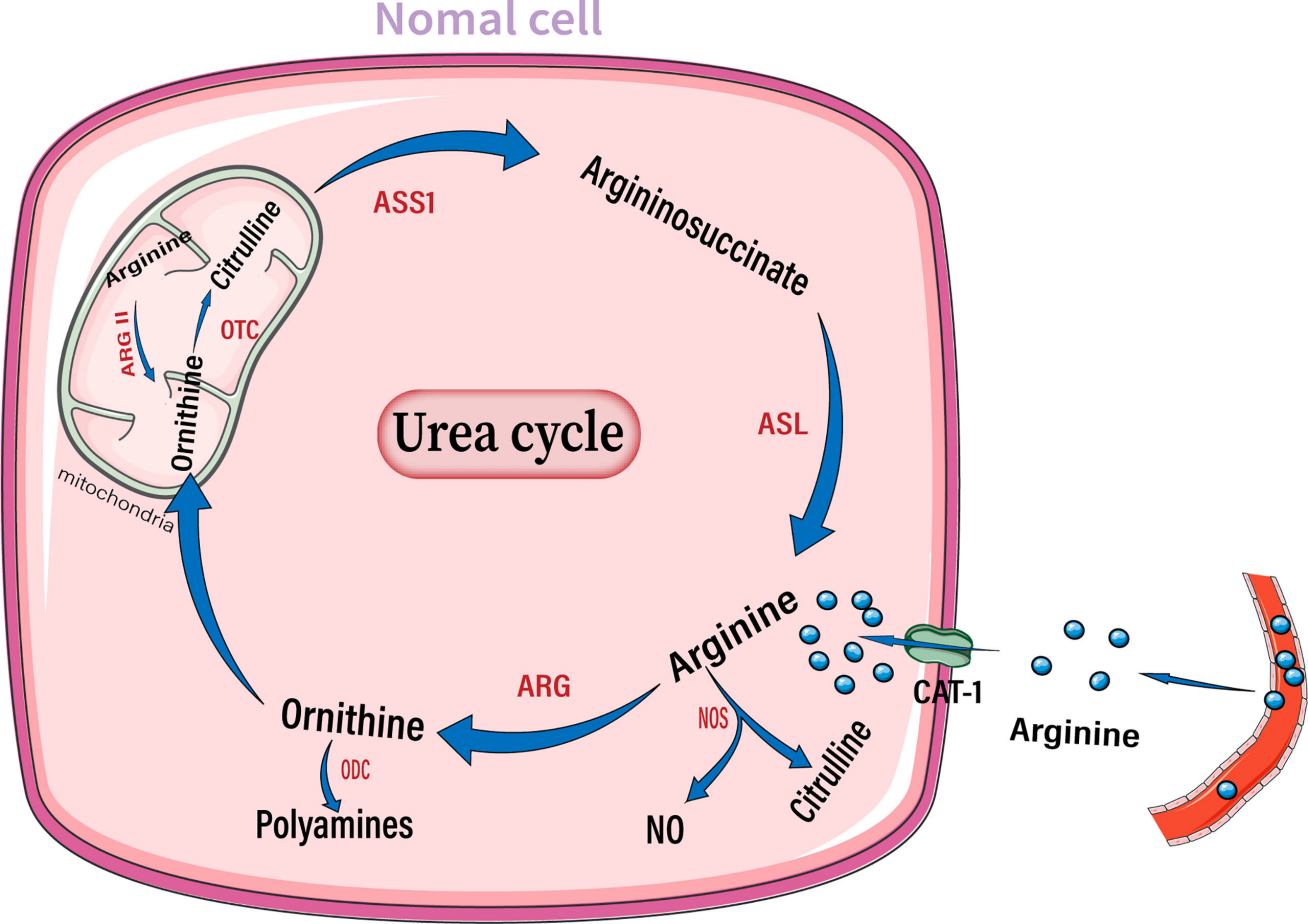
Diagram of the arginine metabolism pattern in normal cells. After entering the extracellular matrix from the circulation, arginine enters the cell through the CAT-1 transporter on the cell membrane. Arginine can be broken down by NOS into NO and citrulline, or be broken down by arginase into ornithine, thus entering the urea cycle. Ornithine can also generate polyamines through ODC. Arginase II in mitochondria is also involved in the degradation of arginine. NO, Nitric oxide; NOS, nitric oxide synthase; ARG, arginase; ODC, ornithine decarboxylase; OTC, ornithine transcarbamylase; ASS1, argininosuccinate synthase; ASL, argininosuccinate lyase.

The intracellular arginine concentration is much higher than the extracellular or plasma arginine concentration. The arginine transporter in most cells is CAT-1, which transports arginine into cells to form the arginine pool. Several enzymes can break down arginine, including arginase, nitric oxide synthase (NOS), arginine decarboxylase, and arginine: glycine amidinotransferase (**Figure 1**) (33, 59).

Quantitatively, arginase is the most important enzyme for arginine decomposition in the body (60). Intracellular arginase hydrolyzes arginine to urea and ornithine. There are two arginase isoenzymes in humans, arginase 1 (Arg1) and arginase 2 (Arg2). Arginase 1 is located in the cytoplasm, its expression is restricted to specific cell types. Moreover, it is transcriptionally regulated by cytokines. Arginase 2 is primarily located in the mitochondria and exhibits a more ubiquitous and constitutive expression pattern, independent of cytokine regulation (61, 62). Ornithine is a metabolite of arginine. Ornithine can enter the urea cycle and is converted to citrulline by ornithine transcarbamylase (OTC). Citrulline synthesizes argininosuccinate through ASS1, which in turn synthesizes arginine through ASL, thus repeating the urea cycle. Ornithine can also generate polyamines *via* ornithine decarboxylase (ODC). Polyamines, including putrescine, spermine, and spermidine, are important products of the arginase metabolic pathway and have tumor-promoting effects (60, 63).

NOS is another important enzyme that breaks down arginine. It breaks down arginine to produce nitric oxide (NO) and citrulline. Notably, arginine is the only substrate for NO production (64). Intracellular arginine increases NO production in a dose-dependent manner (65). There are three distinct isoforms of nitric oxide synthase in the body: NOS1, NOS2, and NOS3, which are encoded by different genes. NOS1 and NOS3 are constitutively expressed in neural and endothelial cells, respectively. NOS2 is a ubiquitous isoform in immune cells, but is not constitutively expressed. Instead, its expression is induced by lipopolysaccharide and inflammatory cytokines; thus, it is called inducible NOS. The roles of NO in tumors are conflicting and may depend on the concentration of NO, type of effector cells, and duration of exposure (66). In general, low concentrations of NO may promote carcinogenesis, cancer cell proliferation, and tumor angiogenesis (67). However, high concentrations of NO can exert cytotoxic effects on tumor cells by inducing DNA damage (68). The complex role of NO in tumors suggests that a comprehensive evaluation of the effect of NO on tumors *in vivo* is essential when targeting arginine metabolism for the treatment of gliomas.

## Reprogramming of Arginine Metabolism in Glioma

**Figure 2:**
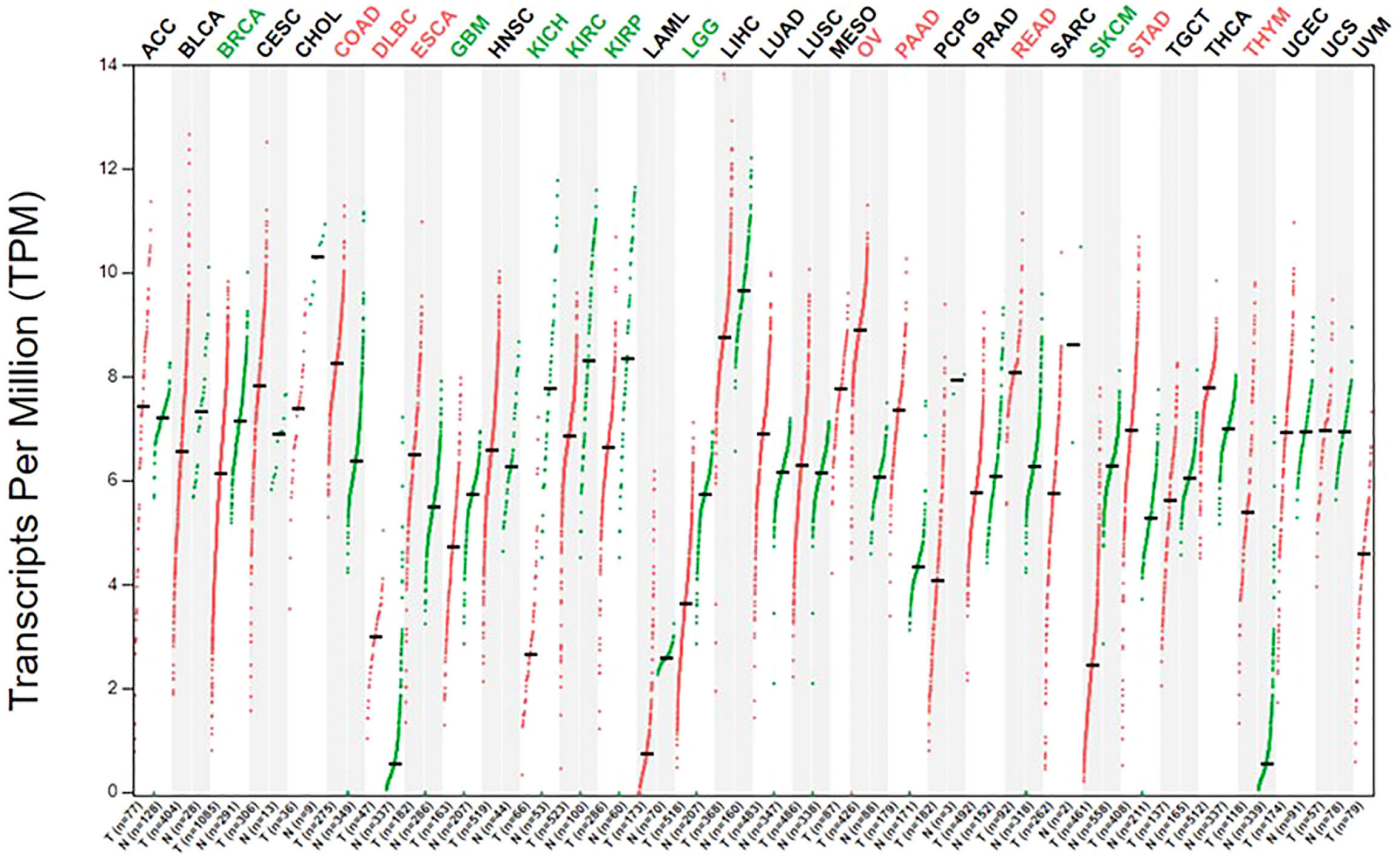
The expression of ASS1 in human normal tissue and cancer cells.The gene expression profile across all tumor samples and paired normal tissues. The figure was excerpted from GEPIA2 (http://gepia2.cancer-pku.cn/#index).

**Figure 3:**
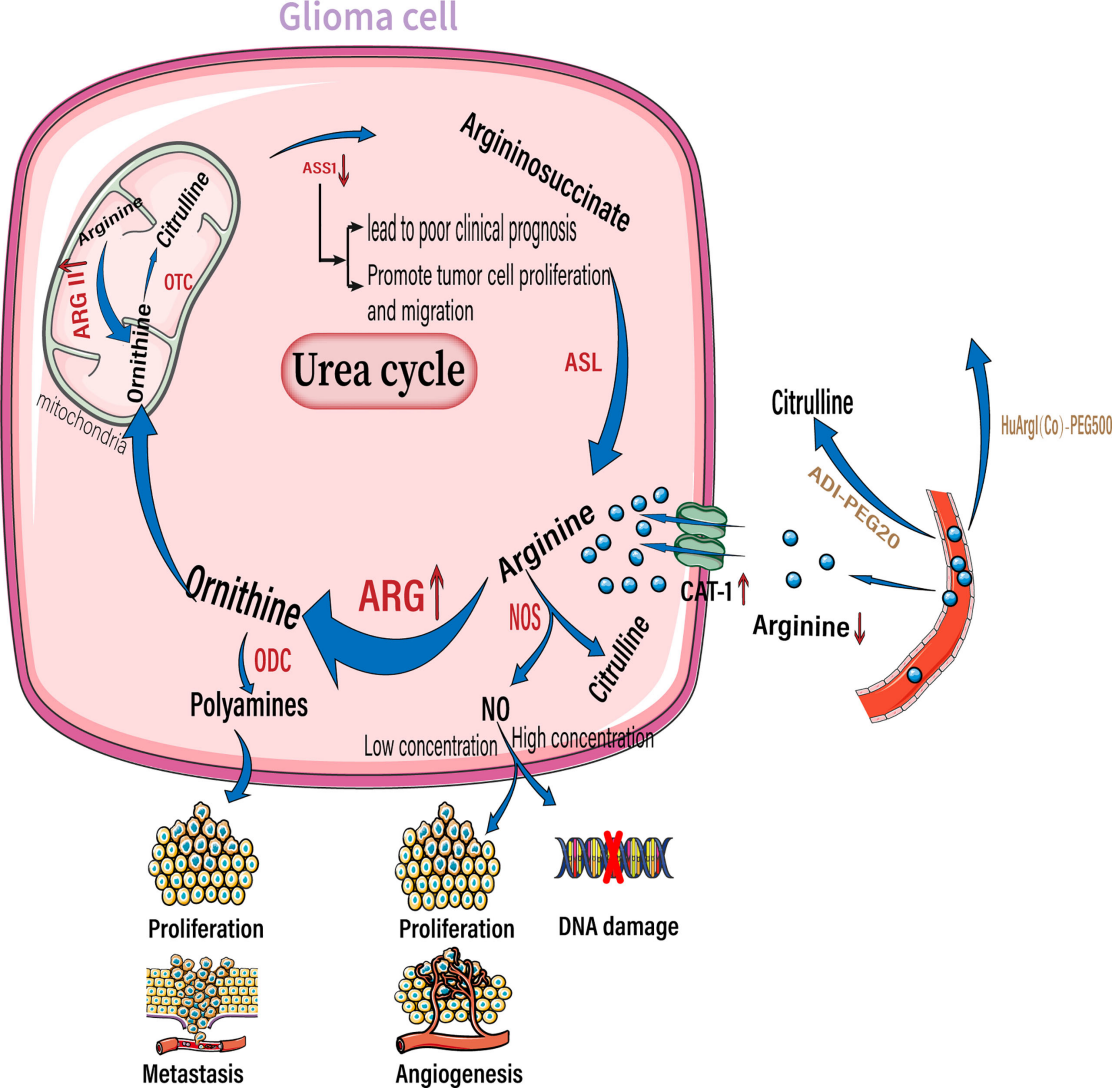
Arginine reprogramming in glioma cells. In glioma cells, ASS1 expression was downregulated while CAT-1 and arginase were upregulated. The upregulated arginase is mainly arginase II located in the mitochondria. The use of ADI-PEG20 and HuArgI(CO)-PEG5000 to break down Circulating arginine results in a significant decrease in arginine concentration in the extracellular environment. Among the downstream metabolites of arginine, polyamines can promote tumor proliferation and metastasis, low concentrations of NO promote tumor proliferation and angiogenesis, and high concentrations of NO cause DNA damage. NO, nitric oxide; NOS, nitric oxide synthase; ARG, arginase; ODC, ornithine decarboxylase; OTC, ornithine transcarbamylase. ASS1,, argininosuccinate synthase; ASL, argininosuccinate lyase.;ADI-PEG20, pegylated arginine deaminase; HuArgI(CO)-PEG5000 ,Pegylated recombinant human arginase I.

Healthy adults obtain arginine primarily through dietary intake and intracellular protein degradation but can also synthesize arginine when needed. This is sufficient to meet the body’s general arginine requirements (69). However, owing to metabolic reprogramming, cancer cells have a greater demand for arginine and rely on the extracellular pool of arginine to sustain their growth (70, 71). Moreover, ASS1 and ASL are downregulated in cancer cells, resulting in the inability to synthesize endogenous arginine, which makes cancer cells more dependent on the extracellular arginine pool (21, 72). This has laid the foundation for arginine deprivation therapy. The expression of ASS1 is varied in different types of tumors; further, the expression of ASS1 is heterogenous even within the same tumor, reflecting tumor heterogeneity (**Figure 2**). In the case of gliomas, 30% of GBM cell lines lack ASS1 expression (**Figure 3**) (73). In general, the downregulation of ASS1 is mediated by promoter methylation or hypoxia-inducible factor (HIF) 1α in multiple cancers. ASS1 levels in cancer are differentially regulated under various environmental conditions to metabolically benefit cancer progression. For example, ASS1 is downregulated under acidic conditions, and ASS1-depleted cancer cells maintain a higher intracellular pH, depend less on extracellular glutamine, and display higher glutathione levels. Cancer cells in an acidic or hypoxic environment downregulate the expression of the urea cycle enzyme ASS1, which provides them with redox and pH advantages, resulting in better survival (74). In response to genotoxic stress, p53 directly promotes ASS1 expression, resulting in increased ASS1 activity. P53-mediated ASS1 induction is a systemic response to genotoxic stress, which can lead to the rearrangement of arginine metabolism at the organism level, as seen in mice (75). Additionally, proline, creatine, and metabolites related to the arginine synthesis pathway were upregulated in ASS1-positive GBM cells compared to ASS1-negative cells. Pyruvic acid, citric acid, and α-ketoglutaric acid are metabolites in the initial phase of the citric acid cycle and are decreased in ASS1 positive cell lines (32). Similarly, tumor cells resistant to the arginine deprivation agent ADI-PEG20, which had upregulated ASS1 expression compared with sensitive cells, showed enhanced expression of glucose transporter-1 and lactate dehydrogenase-A, reduced expression of pyruvate dehydrogenase, and elevated sensitivity to the glycolytic inhibitors, 2-deoxyglucose and 3-bromopyruvate, consistent with the enhanced glycolytic pathway (the Warburg effect). Simultaneously, these cells showed higher glutamine dehydrogenase and glutaminase expression (76). Furthermore, activity-based proteomic profiling and phosphoproteomic profiling were performed before and after ADI-PEG20 treatment of ADI-PEG20-sensitive and -resistant sarcoma cells. Proteomic changes that facilitate oxaloacetate production by enhancing glutamine and pyruvate anaplerosis and altering lipid metabolism to recycle citrate for oxidative glutaminolysis have been elucidated (77). However, whether alterations in these metabolites affect the biological characteristics of gliomas is unclear. However, there is evidence that ASS1 may act as a tumor suppressor gene. For example, patients with GBM lacking ASS1 expression have worse prognosis than ASS1-positive patients (32). Consistent with this finding, decreased ASS1 levels were also significantly associated with postoperative lung metastases and poor clinical outcomes in patients with osteosarcoma. In preclinical studies, overexpression of ASS1 inhibited tumor growth (78). Epigenetic silencing of ASS1 can stimulate tumor cell proliferation and migration (79). These results suggest that ASS1 is a tumor suppressor gene (80). Interestingly, ASS1 may have opposite effects on other tumors. For example, the expression of ASS1 in gastric cancer can promote the invasion of cancer cells, resulting in poor prognosis in patients with gastric cancer (81, 82). Additionally, high ASS1 levels are an indicator of poor disease-free survival in patients with head and neck cancer (83). The dual role of ASS1 in tumors is not fully understood. However, these findings indicate that it is essential to fully understand the expression of ASS1 and its role, before using arginine deprivation therapy for the treatment of specific tumors. The influence of individual differences and tumor heterogeneity should also be considered. The mechanism of ASS1 downregulation, even though not fully elucidated, is undoubtedly beneficial for tumors if ASS1 acts as a tumor suppressor gene. Recent studies have shown that epigenetic changes in two genes involved in arginine biosynthesis in gliomas, namely CpG island methylation of ASS1 and ASL, lead to decreased protein expression. This results in glioma sensitivity to arginine deprivation therapy (84).

Reprogramming of arginine metabolism in gliomas provides a new approach for targeted therapy. But the downside is that this reprogramming also profoundly affects the infiltrating T lymphocytes. This has often been overlooked by researchers who use arginine deprivation therapy to treat gliomas. However, it is not clear whether adaptive changes in T lymphocytes in an arginine-deficient environment can cause glioma tolerance to arginine deprivation therapy. In the following discourse, we explain how gliomas cause a deficit of arginine in the tumor microenvironment and subsequent immunosuppression.

## Glioma Leads to an Arginine-Deficient Immunosuppressive Microenvironment

### Glioma Leads to an Arginine-Deficient Microenvironment

Solid tumors reside in harsh tumor microenvironments together with various stromal cell types. Tumor cells metabolically coordinate or compete with their “neighbors” to meet biosynthetic and bioenergetic demands, while escaping immunosurveillance or therapeutic interventions. The consumption of essential nutrients by cancer cells directly limits the availability of nutrition to the tumor-killing immune cells; this is observed especially with cytotoxic T cells, leading to impaired antitumor immunity. In addition to rapid proliferation, cancer cells outcompete cancer cells by overexpressing transporters for nutrient uptake, and enzymes for nutrient catabolism (85). By upregulating amino acid transporters, glioma cells take up more arginine from the extracellular environment to meet their own proliferation and metabolism requirements. As their requirement of arginine is more than what they are capable of synthesizing, they are highly dependent on arginine availability in the extracellular environment. Therefore, arginine is an essential amino acid (86, 87). Elevated arginine catabolism is a common feature of the tumor microenvironment. The most important enzyme involved in arginine catabolism is arginase, which converts arginine into urea and ornithine. Arginase expression and activity are increased in patients with cancers including glioma, colon cancer, lung cancer, breast cancer, thyroid cancer, prostate cancer, compared to the surrounding healthy tissues in these patients (**Figure 3**) (88, 89). Arginase II is a major subtype expressed by tumor cells (90, 91). Moreover, arginase II is released from tumor cells, such as acute myeloblastoma, and is present in patient plasma at high concentrations (91). Whether arginase II is released outside the cell depends on the type of tumor, as neuroblastomas do not release free arginase II (90). It is unclear whether glioma cells that highly express arginase II release this enzyme. However, regardless of whether tumor cells release arginase, tumors with high arginase expression lead to local and systemic arginine deficiency. For example, patients with renal cell carcinoma and cervical cancer have a corresponding decrease in plasma arginine concentrations at diagnosis, which leads to a poorer prognosis (92, 93).

The increased uptake of arginine and high expression of arginase by tumor cells results in an immunosuppressive phenotype. As mentioned above, arginine deficiency leads to a series of inhibitory phenotypes such as decreased T-cell activation, impaired proliferation, and cycle arrest through multiple mechanisms. It was found that co-culture of Arg2-expressing cancer cells with T cells was sufficient to induce arginine depletion and lead to impaired T-cell proliferation, decreased IFN-γ release, and PD-1 upregulation (25). Moreover, T-cell and myeloid cell infiltration is reduced in head and neck squamous cell carcinomas with high arginase II expression (94). Likewise, in acute myeloid leukemia with high arginase II expression, the surrounding monocytes were more polarized to M2-like macrophages (91). Conversely, arginine replenishment (95) or the use of small-molecule inhibitors of arginase II (91) can alleviate arginine-deficient immunosuppression and reduce T-cell dysfunction (25).

In addition to tumor cells, immunosuppressive cells expressing arginase 1 form an inhibitory immune barrier. The accumulation of ARG1-expressing immunomodulatory cells, including M2-like tumor-associated macrophages, tolerogenic DCs, MDSCs, and Treg cells, in the tumor microenvironment (TME) may suppress antitumor immunity by degrading arginine, thus limiting the availability of this amino acid to T cells (96, 97). Mouse and human tumor cells can secrete soluble factors, such as GM-CSF and G-CSF, which lead to the recruitment and accumulation of MDSCs (98). In GBM patients, the number of circulating MDSCs with high Arg1 expression increases (99). Overexpression of Arg1 in MDSCs leads to downregulation of the CD3ζ chain, which adversely affects CD4+ and CD8+ T cells (100). Additionally, MDSCs exhibit functional similarities to M2-like macrophages (101), including IL-10, TGF-β, and IDO expression (102). This suggests that immunosuppressive cells are closely linked to arginine metabolism; however, this requires further investigations.

Depleting important nutrients such as arginine is a key strategy for cancer cells to evade immunity. Although many tumors are arginine auxotrophic (21), a large proportion can tolerate a low-arginine state (91, 103). This suggests that there must be a unique mechanism that allows these tumors to tolerate an arginine-deficient environment. These tumor cells can synthesize arginine from citrulline by upregulating ASS1. In the absence of arginine, ASS1 transcription is induced by the binding of ATF4 and CEBPb to the enhancer of ASS1. But in T cells, the situation is completely different. Arginine deficiency leads to chromatin compaction and inhibits histone methylation in T cells, which disrupts the binding of ATF4 and CEBPb to ASS1 enhancers and prevents the transcription of target genes (104). These findings help explain the differences in arginine metabolism between tumor cells and T cells and can aid in the development of more effective targeted therapies for the treatment of gliomas.

### Arginine Deficiency Suppresses the Antitumor Function of T Lymphocytes

**Figure 4:**
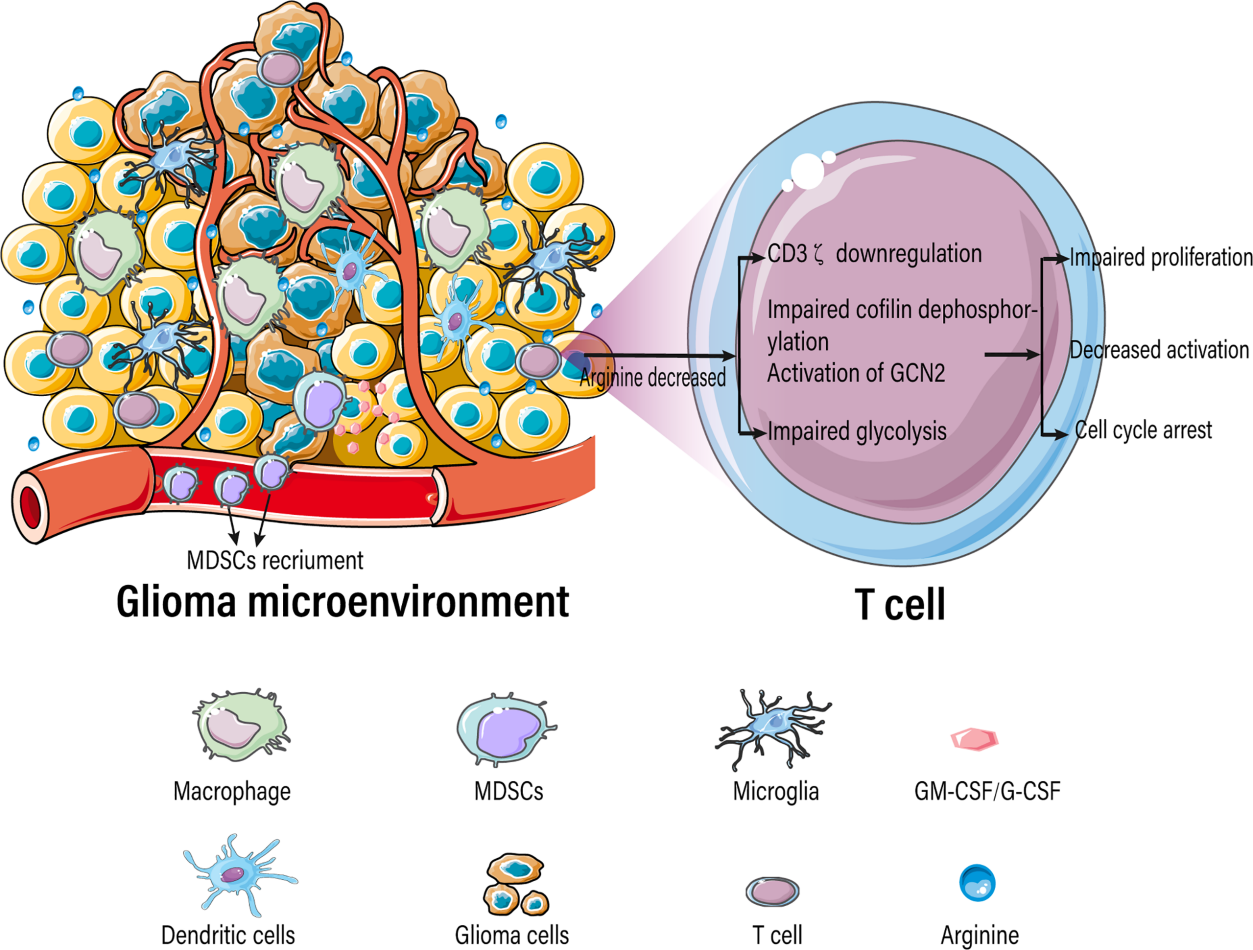
Arginine-deficient glioma microenvironment suppresses T-cell function.

Tumor-infiltrating immune cells typically experience metabolic stress as a result of the dysregulated metabolic activity of tumor cells, leading to impaired antitumor immune responses. Activated T cells consume a large amount of arginine and rapidly convert it into downstream metabolites, resulting in a significant decrease in intracellular arginine levels. T cells are extremely sensitive to extracellular concentrations of arginine because of their low or absent expression of arginine synthase ASS1 and OTC (105, 106). Various studies have demonstrated that arginine deficiency leads to decreased T-cell activation, impaired proliferation, cycle arrest, decreased cytokine (IFN-γ) release, and increased expression of immunosuppressive molecules (PD-1) (**Figure 4**) (22–25). The low arginine levels in the TME also impairs the proliferation of chimeric antigen receptor T cells (CAR-T), limiting their therapeutic effects (107).

In contrast to the lack of arginine, high arginine levels can increase the antitumor activity of T cells, which may be due to a combination of phenotypic alterations, including increased T-cell viability, improved metabolic adaptability, and maintenance of a central memory-like phenotype (95). Therefore, sufficient extracellular arginine is critical for T-cell function. Researchers have exploited the beneficial effects of arginine on T-cell survival and antitumor function to improve adoptive T-cell therapy. For example, CAR-T cells have been reconstituted to express the enzymes ASS1 and OTC, which are required for arginine synthesis. This increases the arginine content in CAR-T cells, thus enhancing the activity of CAR-T cells *in vivo* against solid and hematological tumors (107).

Arginine deficiency-mediated suppression of T-cell function is caused due to a myriad of factors including downregulation of the CD3ζ subunit of the T-cell receptor complex (108–111), damage to cofilin dephosphorylation (112), blockade of protein translation by activation of general control nonderepressible 2 (GCN2) (113), blockage of glycolysis (114), decreased expression of early T-cell activation markers CD25 and CD69 (115), and aberrant expression of D-type cyclins (22, 116, 117).

It is important to note that most studies on the effects of arginine on T cells are based on interventions in extracellular arginine concentrations. For example, change in the concentration of arginine in the T-cell medium. However, extracellular and intracellular arginine pools are not freely interchangeable (118), which means that extracellular arginine supply may not be a reliable indicator of intracellular arginine availability. Recent studies on Arg2 in T cells further demonstrated that the intracellular metabolism of arginine profoundly alters T-cell function. Pharmacological inhibition of arginase increases activation and survival of human T cells *in vitro*. Since human T cells express ARG2, but not ARG1, this suggests that such effects are caused by Arg2 (48). Studies have also found that deletion of Arg2 germline and adoptive transfer of Arg2−/− CD8+ T cells significantly reduced tumor growth in preclinical cancer models by enhancing CD8+ T-cell activation, cytotoxic function, and persistence (48, 119). Importantly, these experiments were performed under arginine excess conditions and, therefore, did not depend on extracellular arginine availability. This indicates that the observed changes are mainly caused by a cellular autonomous mechanism, and that we should focus on the direct effects of intracellular arginine pools on T-cell functions.

## Arginine Replenishment Therapy for Glioma

Researchers have attempted arginine replenishment therapy to treat tumors, by increasing the availability of arginine to improve antitumor immunity. One study found that oral administration of arginine and an anti-PD-L1 antibody restricted tumor growth and increased survival in mice, suggesting a synergistic effect of arginine and PD-L1 blockers. To achieve the desired antitumor effect, mice must be administered a relatively high dose of arginine (2 mg/g of body weight). In comparison, an adult patient weighing 75 kg would require 150g of arginine per day, which is unrealistic. Therefore, researchers have developed metabolically engineered bacteria called L-Arg bacteria, to be planted in the tumors, which will produce large amounts of arginine. L-Arg bacteria and PD-L1 blockers can synergistically inhibit tumor growth, increase the infiltration of CD4+ and CD8+ T cells, and reduce the infiltration of Treg cells in the tumor. Further studies also found that this combination reduced the percentage of PD-1+LAG-3+ T cells, indicating the persistence of effector T-cell function with the simultaneous increase in the formation of tumor-specific memory T cells (120). Similar studies have found that arginine increases radiosensitivity in patients with brain metastases. Additional oral administration of arginine before standard radiotherapy in 31 patients with brain metastases significantly improved the therapeutic effect of radiotherapy. This therapeutic effect is due to NO-induced metabolic inhibition, which increases the susceptibility of NOS2-expressing cancer cells to DNA damage (121). NOS2 activity is required for tumor brain metastasis and it can decompose arginine to NO. Arginine increases radiosensitivity through an NO-mediated mechanism, and high intratumoral NO concentrations lead to a decrease in tumor glycolysis and thus a decrease in lactate levels. These metabolic changes ultimately impair the repair of radiation-induced DNA damage in cancer cells. In addition, the authors suggested that the enhanced overall antitumor effect may also be due to immune activation. In mouse tumor models, oral administration of arginine improved the metabolic adaptability of T cells, which is critical for antitumor responses (95). Administration of arginine prior to radiation therapy reversed radiation-induced T-cell and B-cell dependent immune dysfunction in mice (122). Although this mechanism has not been fully elucidated, it is speculated that arginine-induced reduction in lactate levels may also contribute to the enhanced antitumor activity of tumor-infiltrating lymphocytes (123).

## Arginine Deprivation Therapy for Glioma

Arginine deprivation therapy is a novel antimetabolic strategy that exploits the differential expression of key urea cycle enzymes to treat arginine auxotrophic tumors. Arginine deaminase (ADI), a metabolic enzyme extracted from Mycoplasma (124), catalyzes the conversion of arginine to citrulline. Owing to its instability, strong immunogenicity, and short half-life (5 h), ADI is combined with polyethylene glycol (ADI-PEG20) to reduce antigenicity and prolong half-life (125). Synthetic human arginase 1 (HuArgI) is another arginine deprivation agent used to treat arginine auxotrophic tumors. Its activity is also enhanced by adding polyethylene glycol and replacing Mn2+ with Co2+, resulting in HuArgI(CO)-PEG5000. HuArgI(CO)-PEG5000 lasts longer in serum, has better catalytic activity, and is less exposed to the immune system (126–128).

If the cells were not rescued by adding citrulline after arginine depletion, these cell lines were completely auxotrophic to arginine; however, when rescued after adding citrulline, the cell lines became partially auxotrophic. Pegylated recombinant human arginase I was used to target nine GBM cell lines and human fetal glial cells (SVG-p12), and was found to be cytotoxic to all GBM cell lines except SVG-p12 cells, which shows selective cytotoxicity induced by arginine deprivation. Subsequent addition of citrulline rescued these six GBM cell lines. The ability of citrulline to rescue cells was dependent on argininosuccinate synthase 1 expression, and cells that were not rescued were negative for ASS1 expression. Knockdown of ASS1 reversed the ability of citrulline to rescue GBM cells, further illustrating the dependence on ASS1 expression (129). Approximately 30% of GBMs lack ASS1 expression and can be targeted by arginase I, which has no cytotoxicity to normal glial cells. Likewise, depletion of arginine using pegylated arginine deaminase resulted in cell death *in vitro* and tumor regression in orthotopic xenograft models, whereas ASS1-expressing GBM cells were unaffected (84, 130). In addition, researchers also found that the use of arginine deprivation agents in combination with other treatments showed better therapeutic effects. Many studies have described the molecular mechanism of arginine deprivation in ASS1 deficient tumors, thereby uncovering additional vulnerabilities in these tumors. This has prompted the use of other drugs in combination with arginine deprivation therapy for more effective killing of tumor cells. For example, TRAIL is used for mesothelioma (131), cisplatin is used for various tumor types (132), and chloroquine is used for sarcoma (133). The combination of arginine deprivation and canavanine, a plant-derived arginine analog, is a novel approach to glioma treatment. This combination therapy profoundly affects cell viability, morphology, motility, and adhesion. It also disrupts the cytoskeleton and mitochondrial network, thereby inducing apoptosis. At the molecular level, canavanine inhibits pro-survival kinases such as FAK, AKT, and AMPK. Importantly, these effects are selective to GBM cells, as shown by their less pronounced effects on rat glial cells (134). Similarly, the combination of ADI and Palomid 529, an inhibitor of mTORC1 and mTORC2 complexes, showed a potent cytotoxic effect in glioma cell lines. In addition, ADI combined with chloroquine showed an enhanced antitumor effect. *In vivo*, ADI alone and the combination of ADI and SAHA, a protein deacetylase inhibitor, effectively inhibited the growth of xenograft tumors (135). A recent phase I clinical trial preliminarily verified the therapeutic effects of arginine deprivation therapy. Ten patients with severe ASS1-deficient recurrent high-grade gliomas were treated with ADI-PEG20 in combination with pemetrexed and cisplatin. The treatment was safe and well tolerated by the patients. The best overall response was stable disease in eight patients (80%). The results showed that the treatment was well tolerated and 80% of patients had stable overall efficacy, with plasma arginine significantly suppressed below baseline levels. However, the titers of anti-ADI-PEG20 antibodies in patients increased, indicating the production of neutralizing antibodies, which may affect the therapeutic effect of ADI-PEG20 (136). Additional clinical studies on arginine depletion in glioma treatment are presented in **Table 1**.

**Table 1:** Clinical studies on arginine depletion in glioma treatment.

Disease	Treatment	Clinical phase	No. of patients	Status	Clinical Trials.gov Identifier
Recurrent high-grade glioma (ASS1-deficient)	ADI-PEG20 with pemetrexed and cisplatin	Phase I	10	Terminated	NCT02029690
Glioblastoma multiforme	ADI-PEG 20 with Radiotherapy and Temozolomide	Phase I	32 (Estimated)	Recruiting	NCT04587830
Advanced solid cancers	ADI-PEG 20 with pembrolizumab	Phase I	33	Terminated	NCT03254732
High-grade gliomas and others	rhArg1peg5000	phase I/II	64 (Estimated; Children and Young Adults)	Unknown	NCT03455140
Advanced/Metastatic solid tumors	INCMGA00012 (PD-1 Inhibitor), INCB001158(Arginase Inhibitor), and the combination	Phase I	18	Completed	NCT03910530
Advanced/Metastatic solid tumors	INCB001158 with chemotherapy	phase I/II	149	Active, not recruiting	NCT03314935

The above findings suggest that arginine deprivation therapy is only effective in ASS1-negative glioma and has little effect on ASS1-positive glioma (including adaptive transcriptional upregulation of ASS1 after treatment), which greatly limits the clinical applications of ADT. Therefore, researchers have attempted to combine ADT with other treatment modalities to improve the curative effect of the treatment for ASS1-positive gliomas. Animals bearing intracranial human GBM tumors of varying ASS status were treated with ADI-PEG20 alone or in combination with temozolomide and monitored for tumor growth and regression. ADI-PEG20 monotherapy significantly reduces intracranial growth of ASS1-negative GBM and extends survival of mice carrying ASS1 negative GBM without obvious toxicity. ADI-PEG20 combined with temozolomide shows enhanced antitumor effects in both ASS1-negative and ASS1-positive backgrounds. The mechanism underlying this effect is unclear, but these results suggest that ADI-PEG20 in combination with TMZ may be clinically useful in both ASS1-negative and ASS1-positive settings (130). In addition, ADT combined with radiotherapy may be a new treatment strategy for patients with GBM. ADT caused significant radiosensitization, which was more pronounced in a GBM cell model with loss of function of p53 than in its p53- wildtype counterpart. This synergistic effect was independent of basic and induced ASS1 or ASL expression (137). ADI-PEG20 also significantly enhanced the efficacy of radiotherapy for ASS1-positive GBM *in vivo* (73). However, ADT combined with radiotherapy has not yet been studied in clinical trials.

The mechanism of glioma cell death in the absence of arginine has not yet been fully elucidated. GBM exhibits caspase-independent, non-apoptotic cell death upon arginine deprivation. The latter, a process known as autophagy, provides a temporary but limited supply of arginine through the destruction of intracellular organelles. Therefore, this process protects against cell death, but leads to non-apoptotic death in the long run. The autophagy inhibitor, chloroquine, was added to GBM cells treated with HuArgI(CO)-PEG5000. As expected, the inhibition of autophagy increased the sensitivity of cells to HuArgI(CO)-PEG5000 (129, 138). After emphasizing the effect of arginine deficiency on cell viability, it is important to observe the effect of arginine deficiency on cell motility and migration ability. Arginine deficiency affects tumor cell morphology and inhibits motility, invasiveness, and adhesion. Moreover, it has little effect on normal glial cells. This is because of specific changes in actin assembly caused by arginine deprivation in gliomas. Arginine deprivation reduces β-actin filament content and affects N-terminal arginylation. This suggests that arginine deprivation-based therapeutic strategies can inhibit the invasive process of highly malignant brain tumors (139).

The combined treatment with ADI-PEG20 significantly enhanced the efficacy of GBM radiotherapy in a non-arginine auxotrophic background. This combination results in a durable, complete radiological, and pathological response. It also prolonged disease-free survival in an *in situ* model of GBM with no apparent toxicity (73). Further studies found that the combination treatment resulted in downregulation of Arg1 and upregulation of inducible NOS. Under arginine-deficient conditions, inducible NOS has a higher affinity for arginine than for Arg1. Combination therapy increased the production of NO, which further formed cytotoxic peroxynitrite (140). This could enhance the sensitivity of ASS1-positive GBM to ionizing radiation (141). In addition, arginine deficiency greatly reduces vasogenic edema and neovascularization, which are typical features of GBM (142, 143). The antiangiogenic activity of ADI appears to be partly due to the twisting of actin filaments, which prevents blood vessels from sprouting, blooming, and growing. ADI-PEG20 also inhibits HIF, particularly HIF-1α (144). HIF-1α is associated with a decrease in the expression of vascular endothelial growth factor, which induces blood vessel growth. HIF has also been implicated in the pathogenesis of GBM (145). High HIF-1α levels also reduce glioma responsiveness to TMZ (146). Thus, ADI-PEG20 has antitumor effects, at least in part, due to its anti-HIF effects.

The antitumor properties of ADT have been extensively investigated. ADT inhibits the growth of auxotrophic cancers *in vitro* and *in vivo*. However, its impact on immune cells in the tumor microenvironment remains, largely, unknown. The removal of arginine can theoretically impair the immune function of T cells. Interestingly, ADI-PEG20 led to a marked increase in tumor-infiltrating CD4+ and CD8+ T cells in a syngeneic B16-F10-melanoma mouse model (147). Similarly, arginine deprivation combined with radiotherapy increased recruitment of microglia into tumors in a glioma model and enhanced their activity and phagocytic phenotype. Arginine deprivation switched the activation of tumor-associated macrophages/microglia from a tumor-supporting phenotype to a more phagocytosis-competent and, hence, tumor-inhibiting phenotype. Simultaneously, a significant increase in the number of CD4+ and CD8+ T cells and a corresponding decrease in FoxP3+ regulatory cells was observed in the glioma microenvironment (73). Despite the increased number of infiltrating T cells, it remains unknown whether T-cell function is affected. It is important to further explore changes in the tumor immune microenvironment after ADT treatment.

Studies on the potential mechanism of ADT resistance have found that the re-expression of ASS1, production of neutralizing antibodies to arginine deprivation agents, and autophagy are the main causes. ADT, by nutrient starvation or exposure to ADI-PEG20, induces adaptive transcriptional upregulation of ASS1 and ASL in glioma cells *in vitro*, thereby conferring resistance to ADI-PEG20 treatment. The specific mechanism of the adaptive transcriptional upregulation of ASS1 and ASL is unknown, but studies in melanoma suggest that accumulated cMyc can induce ASS1 expression by interacting with the ASS1 promoter (148, 149). Although the modification of ADI by conjugation with polyethylene glycol can reduce its immunogenicity, the production of anti-ADI antibodies has also been observed in patients enrolled in clinical trials. This suggests that long-term treatment may lead to the development of resistance due to the production of neutralizing antibodies. This phenomenon may explain the negative correlation between plasma neutralizing antibody levels and duration of arginine depletion after ADI-PEG20 treatment (136). When arginase I was combined with the autophagy inhibitor, chloroquine, to treat GBM *in vitro*, the inhibition of autophagy increased cellular sensitivity to arginase I. This finding suggested that autophagy plays a supporting role in ADI resistance (129). Arginine deprivation agents for cancer treatment should have low toxicity, non-immunogenicity (to prevent antibody production and allergic reactions), rapid action (to delay the emergence of resistance), and long circulating half-lives (to achieve sustained arginine consumption) (150). It is worth investigating whether low arginine levels during arginine deprivation therapy can adversely affect antitumor immunity, since T-cell function is regulated by arginine. In addition, the reconstruction of adaptive immune function against the background of arginine-mediated tumor immune escape is a promising therapeutic strategy.

Metabolic reprogramming is often mediated by oncogenic signaling pathways. In particular, mTOR signaling is commonly activated in tumors and controls cancer cell metabolism by altering the expression and/or activity of several key metabolic enzymes (151). Conversely, metabolic alterations affect mTOR signaling. mTORC1 is one of the mechanisms that checks cellular amino acid levels and/or nutritional deprivation in cells. For example, arginine activates mTORC1 through the GATOR1/2-Rag pathway by directly binding to the arginine sensor CASTOR1 (Cellular Arginine Sensor for mTORC1) (151). Interestingly, ASS1 knockdown results in increased mTORC1 activity in osteosarcoma cells, potentially due to increased aspartate levels (86). Treatment with rhARG reduces mTORC1 activity and induces cytotoxicity and apoptosis in non-SCLC cells (152). However, resistance to arginine deprivation agents has been observed. ADI-PEG20-resistant tumor cells exhibited reduced mTOR signaling but enhanced AKT signaling, which led to the stabilization of MYC. MYC in turn induces ASS1 expression by competing with HIF1α for ASS1 promoter-binding sites (76). The molecular mechanism underlying the downregulation of mTOR signaling in ADI resistance remains unclear.

## Advantages and Disadvantages of Targeting Arginine Therapies for Glioma

We now summarize the advantages and disadvantages of Arginine deprivation therapy and Arginine replenishment therapy as follows:

Advantages of Arginine deprivation therapy (1): There are mainly five enzymatic agents catabolizing free arginine in theory (NOS, glycine amidinotransferase, arginine decarboxylase, arginase, and arginine deiminase) (153). This provides a variety of options for arginine deprivation therapy (2). Arginine deprivation therapy achieves its therapeutic effect by lowering the plasma arginine concentration, which is especially appropriate for intracranial tumors and is no longer hindered by the blood-brain barrier (3). Arginine deprivation therapy has completed different clinical trials in patients with metastatic melanoma and mesothelioma with promising results (154, 155) (4). Arginine deprivation therapies have different mechanisms in tumors, such as induction of autophagy, ROS overproduction, cell cycle arrest, and caspase-dependent/independent apoptosis in cells. Thus, AD therapy has the potential to treat tumors in combination with other treatments.

Disadvantages of Arginine deprivation therapy (1): The resistance of tumors to arginine deprivation agents is currently the biggest obstacle, mainly due to the re-expression of ASS1, production of neutralizing antibodies to arginine deprivation agents, and autophagy. We urgently need to elucidate the underlying mechanisms of drug resistance to increase their therapeutic efficacy against tumors (2). The therapeutic effect of arginine deprivation depends largely on whether the tumor is auxotrophic. In other words, it depends on the expression of ASS1 in tumor cells. This greatly limits the application of arginine deprivation agents. However, there are ongoing research studies to circumvent this problem. For example, a combination of arginine deprivation therapy with radiotherapy or TMZ has shown a good therapeutic effect on ASS1-positive gliomas.

Advantages of Arginine replenishment therapy: Arginine is an inexpensive, readily available amino acid that cancer patients only need to consume orally. This greatly increases the convenience of this treatment. Moreover, arginine is a nutrient needed by the body and does not produce toxic side effects like other chemotherapeutic drugs.

Disadvantages of Arginine replenishment therapy: Arginine replenishment therapy requires a high concentration of arginine in the tumor microenvironment in order to achieve a good therapeutic effect. Achieving the required concentration poses a challenge that needs to be addressed urgently.

## Discussion

The advantages of targeting arginine in the treatment of gliomas are evident. It kills tumor cells directly or indirectly by interfering with tumor cell metabolism, without affecting normal cell function. Concurrently, it can bypass the blood-brain barrier, which is especially suitable for intracranial diseases. Arginine deprivation therapy works directly on tumor cells. The combination of arginine deprivation with other treatments has shown great potential and application value, and requires further in-depth research. Arginine replenishment therapy is more likely to act on immune cells and affect tumor cells, which is an indirect mechanism. Although the two treatments seem contradictory, differences in their mechanisms of action make us interested in finding ways to combine them. Currently, targeting arginine metabolism to treat glioma faces the dilemma of choosing arginine deprivation therapy or arginine replenishment therapy. The former achieves tumor inhibition by “starving” tumor cells, but its negative effects are often ignored by researchers. The arginine-deficient extracellular environment created by arginine deprivation agents undoubtedly exerts a strong inhibitory effect on antitumor T cells. Further studies are required to determine whether the suppressed T cells are responsible for the poor effects of arginine deprivation therapy. The latter increases the antitumor activity of T cell by fulfilling their arginine requirements. Likewise, the arginine replenishment therapy “feeds” tumor cells. The direct effect of excess arginine on glioma cells is unclear, but we do not want tumor cells to be “nutrient-rich.” Another strategy to increase arginine levels in the body is to prevent its breakdown. In mouse tumor models, ARG1 inhibitors, which prevent the breakdown of arginine, increase CD8+ T-cell infiltration and stimulate the production of inflammatory cytokines in the TME (97, 156). Further studies are needed to determine the therapeutic effect of ARG1 inhibitors on glioma. Most existing studies describe arginine deprivation therapy as the chosen method to treat brain tumors; however, a few studies have also described arginine replenishment therapy to treat brain tumors. Here, we hope to adopt a suitable strategy to combine the two strategies, both “starving” tumor cells and enhancing antitumor immune response. CAR-T therapy combined with arginine deprivation therapy may be an effective strategy to circumvent this pitfall. CAR-T cells can recombinantly express ASS1 and OTC, increasing the arginine content in cells. This increases the persistence of CAR-T cells *in vivo* (107). However, we still need to conduct extensive preclinical studies to determine the effectiveness of this therapy. We hope that this will open new avenues for comprehensive treatment for glioma.

## Author Contributions

XH and BW contributed to the conceptual design, and helped in the writing and editing of the manuscript. SC, PZ, and DG revised the manuscript and commented on its previous versions. All authors contributed to the article and approved the submitted version.

## Funding

This work was supported by the National Natural Science Foundation of China (Grant No. 82072797), the National Natural Science Foundation of China (Grant No. 81874086), and Hubei Provincial Natural Science Foundation of China (Grant No. 2020CFB671)

## Conflict of Interest

The authors declare that the research was conducted in the absence of any commercial or financial relationships that could be construed as a potential conflict of interest.

## Publisher’s Note

All claims expressed in this article are solely those of the authors and do not necessarily represent those of their affiliated organizations, or those of the publisher, the editors and the reviewers. Any product that may be evaluated in this article, or claim that may be made by its manufacturer, is not guaranteed or endorsed by the publisher.
